# Bulbar conjunctival metastasis from mucoepidermoid carcinoma of parotid—a case report and review of literature

**DOI:** 10.1186/s12957-016-1077-0

**Published:** 2017-01-07

**Authors:** Rajshri Yadav, Azhar J. Battoo, Abdul W. Mir, Altaf G. Haji

**Affiliations:** 1 Department of Otorhinolaryngology, Sher-i-Kashmir Institute of Medical Sciences Medical College, Srinagar, 190011 India; 2 Surgical Oncology, Sher-i-Kashmir Institute of Medical Sciences, Srinagar, 190011 India; 3 Department of Surgical Oncology, Sher-i-Kashmir Institute of Medical Sciences, Srinagar, 190011 India

**Keywords:** Bulbar conjunctiva, Distal metastasis, Mucoepidermoid carcinoma, Parotid, Case report

## Abstract

**Background:**

Mucoepidermoid carcinoma of salivary glands usually metastasizes to the lungs, liver, bone, brain, and skin. We report a rare case of distant metastasis of high-grade mucoepidermoid carcinoma of the parotid to the ipsilateral bulbar conjunctiva of the eye.

**Case presentation:**

Sixty-year-old male of Kashmiri origin presented to our tertiary care referral cancer institute with exophytic lesion of the left bulbar conjunctiva following his treatment for mucoepidermoid cancer of ipsilateral parotid gland, 9 months back. The lesion was biopsied and reported as high-grade mucoepidermoid carcinoma. Radiological imaging showed no other site of recurrence. The patient underwent orbital exenteration and final histopathological evaluation reported the lesion as mucoepidermoid carcinoma.

**Conclusions:**

Distal metastasis from mucoepidermoid carcinoma to bulbar conjunctiva is very rare and to the best of our knowledge has not been previously reported.

## Background

The incidence of distant metastasis seen in mucoepidermoid carcinoma (MEC) of salivary glands is almost 13% [1]. Distant metastasis is most commonly seen in the lung (25%) followed by the liver (25%) and bone (18%), respectively [2].

We report here a rare case of isolated distal metastasis of MEC parotid to the bulbar conjunctiva of the ipsilateral eye. The consent for publishing this case report was taken from the institute’s ethics committee—Sher-i-Kashmir Institute of Medical Sciences Ethics Committee. Such a case is unusual and, to our knowledge (following thorough search on PubMed and Google), has not been previously reported.

## Case presentation

A 65-year-old Kashmiri male patient, hypertensive, presented to the Surgical Oncology department of a tertiary care referral center with exophytic lesion of the left bulbar conjunctiva, following his initial surgery for left parotid gland mucoepidermoid carcinoma. Magnetic resonance imaging of the head and neck along with computed tomographic scan of the chest was done before the previous surgery, which showed lesion confined to the parotid gland, without any regional or distant metastases. The patient underwent radical parotidectomy along with neck dissection followed by radiotherapy. Histopathological examination of the operated specimen had showed features of high-grade MEC (Brandwein’s modified AFIP criteria; score 8), and patient was staged as pT4N0M0. After almost 10 months of the surgery, the patient developed visual impairment in the left eye for which he consulted the ophthalmologist, where on examination, lateral bulbar conjunctival growth was seen. The lesion was biopsied and histopathology revealed features of high-grade MEC.

**Fig. 1:**
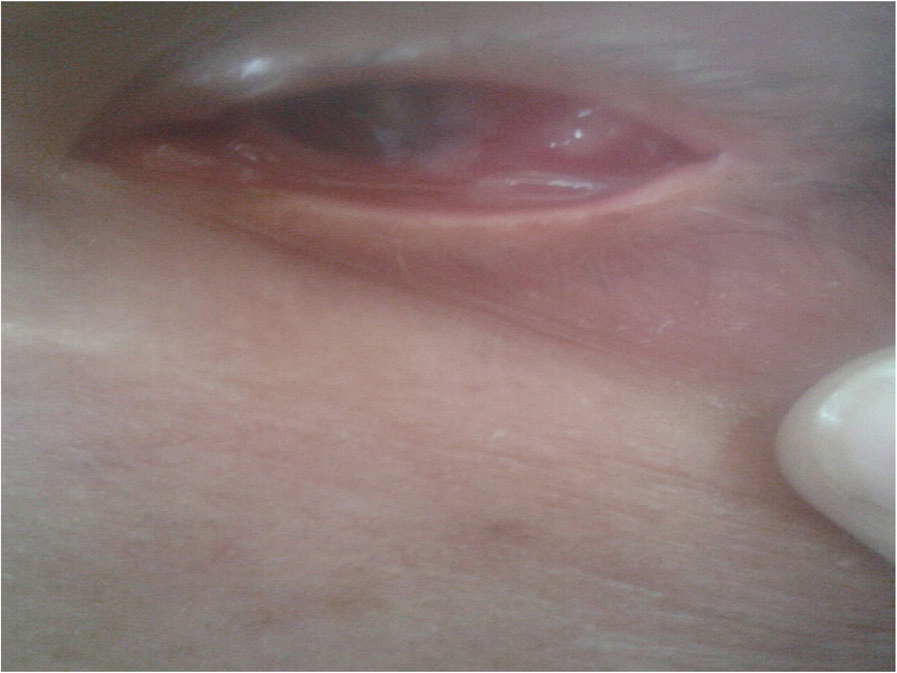
Preoperative appearance of orbital lesion, showing exophytic growth involving lateral bulbar conjunctiva

Examination of patient revealed exophytic growth involving mainly lateral aspect of the bulbar conjunctiva. Inferior palpebral fissure was obliterated by the growth; superior palpebral fissure was free. Corneal opacity seen on lateral aspect extended up to the pupil. Both the upper and lower eyelids were thickened (Fig. 1).

**Fig. 2:**
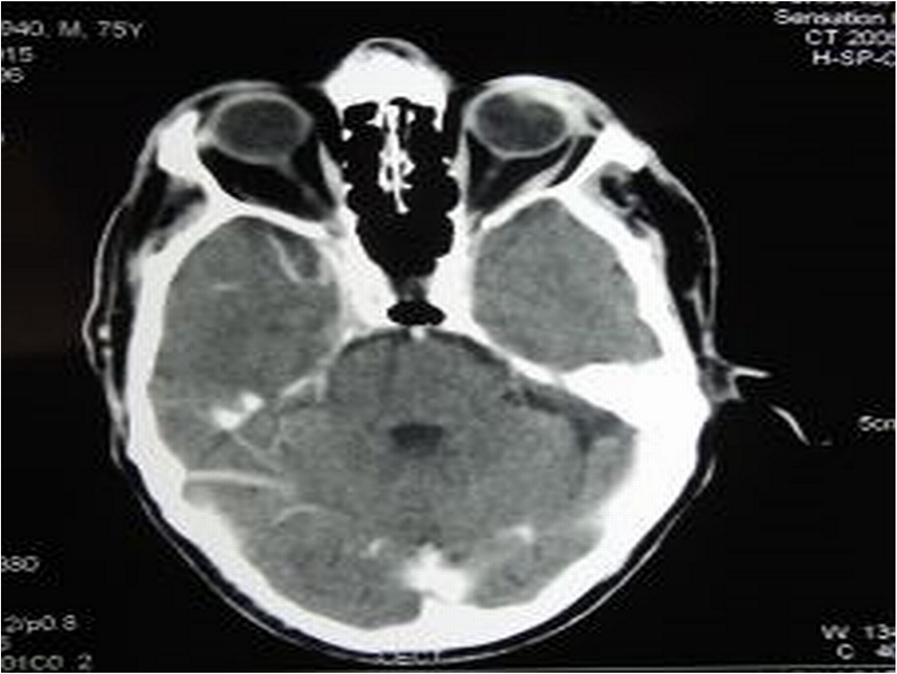
Preoperative contrast enhanced computed tomography of patient showing enhancing lesion involving anterolateral wall of the left eyeball and thickened overlying eyelid

Contrast enhanced computed tomography of the brain, neck, and chest revealed evidence of enhancing lesion involving anterolateral wall of the left eyeball and overlying eyelid appeared thickened with no extension into the post-septal compartment (Fig. 2). No evidence of residual or recurrent mass lesion in the left parotid region was seen. No other evidence of loco regional or distant metastasis was seen.

The case was discussed in the Institution’s Tumour Board, and patient was planned for left orbital exenteration followed by split thickness skin graft lining for orbital cavity. The patient was explained about the prognosis of the disease, and consent for the procedure was taken. The left orbital exenteration was done along with the excision of the upper and lower eyelids. Split thickness skin graft was harvested from the thigh and grafted at the site of defect.

**Fig. 3:**
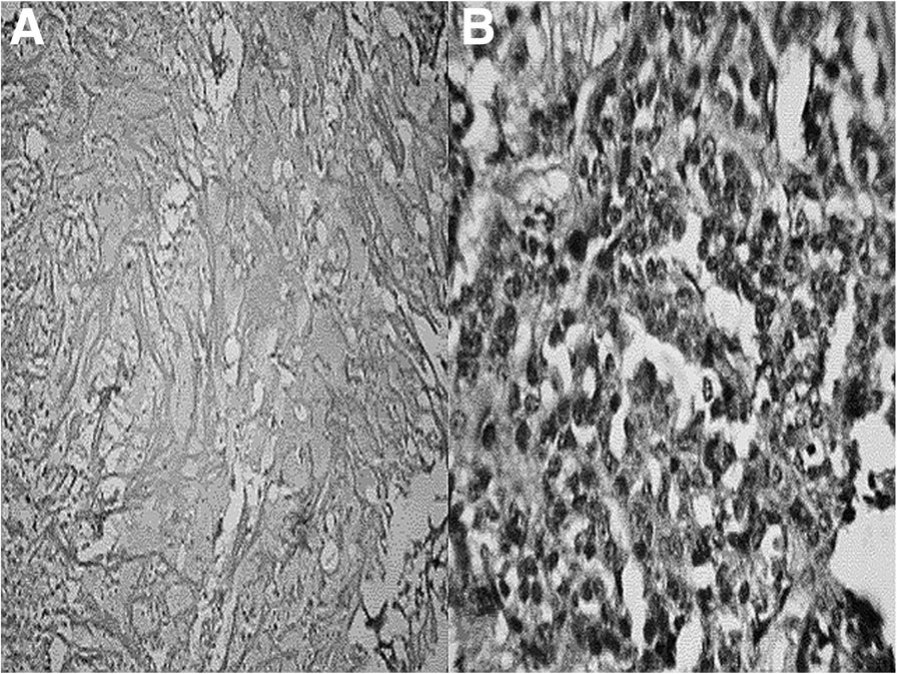
Hematoxylin and eosin staining (original magnification ×40) showing **a** areas of extracellular mucin and **b** tumor cells showing squamoid differentiation

Histopathological examination of the specimen showed features of mucoepidermoid carcinoma infiltrating up to the sclera, high-grade type (Brandwein’s modified AFIP score 7). Infiltration was also seen in the lower eyelid. All resection margins were free of tumor, greater than 5 mm. Optic nerve resection margin was free of tumor (Fig. 3a, b). Post-surgery patient did well with no evidence of loco regional or any other distant recurrence at the time of writing the article.

### Discussion

The aggressiveness of mucoepidermoid carcinomas is graded as per histological grading system introduced by Brandwein et al. [3], which is based on the eight components namely intracystic component <25% (+2), aggressive pattern of invasion (+2), anaplasia (+2), perineural invasion (+3), necrosis (+3), >4 mitosis/ HPF (high-power field) (+3), bony invasion (+3), and lympho-vascular invasion (+3). Scoring for low-grade tumors is 0, for intermediate grade tumors 2 and 3, and for high-grade tumors >4. The microscopic examination of the specimen obtained after orbital exenteration in our patient showed features of extensive necrosis (+3), cystic component <25% (+2), and anaplasia (+2), which made a total score of 7, consistent with high-grade MEC. Extravasated mucin was also seen which is an evidence of the aggressive nature of the tumor.

Chen et al. [4] evaluated medical records of 61 patients of parotid gland MEC. A multivariate analysis of the entire patient sample revealed high-grade tumor and T4 disease as independent predictors of decreased survival in that order (LLR test: *P* = 0.0001 and 0.02, respectively). Out of 61 patients, 20 developed distant metastases, 14 of which were isolated events. Initial sites of distant failure were the following: 16 lungs, 3 bones, and 1 liver. Median time for development of distant failure was 20 months (range, 6–70). High histologic grade and pathological lymph node metastasis was associated with a sufficiently greater risk of distant metastasis. The 5-year distant metastasis-free survival was 87% for patients, with non-high-grade tumors compared to 47% for those with high-grade tumors (*P* = 0.001). The 5-year estimate of distant metastasis-free survival for patients with and without pathological lymph node metastasis was 57 and 80%, respectively (*P* = 0.03). The patient in consideration of the present case report also had T4 disease with invasion into the masseter muscle and high-grade tumor on histopathology.

## Conclusions

To conclude high-grade and T3–T4 MEC, tumors have aggressive behavior and propensity for distant metastasis but metastasis to bulbar conjunctiva is extremely rare and to the best of our knowledge has never been reported.

## Abbreviations

MEC: Mucoepidermoid carcinoma
